# The Effect of Heat Transfer and Polymer Concentration on Non-Newtonian Fluid from Pore-Scale Simulation of Rock X-ray Micro-CT

**DOI:** 10.3390/polym9100509

**Published:** 2017-10-13

**Authors:** Moussa Tembely, Ali M. AlSumaiti, Mohamed S. Jouini, Khurshed Rahimov

**Affiliations:** Petroleum Institute, Khalifa University of Science and Technology, P.O. Box 2533, Abu Dhabi, UAE; aalsumaiti@pi.ac.ae (A.M.A.); mjouini@pi.ac.ae (M.S.J.); khrahimov@pi.ac.ae (K.R.)

**Keywords:** pore-scale model, non-Newtonian fluid, Finite Volume Method, Digital Rock Physics

## Abstract

Most of the pore-scale imaging and simulations of non-Newtonian fluid are based on the simplifying geometry of network modeling and overlook the fluid rheology and heat transfer. In the present paper, we developed a non-isothermal and non-Newtonian numerical model of the flow properties at pore-scale by simulation of the 3D micro-CT images using a Finite Volume Method (FVM). The numerical model is based on the resolution of the momentum and energy conservation equations. Owing to an adaptive mesh generation technique and appropriate boundary conditions, rock permeability and mobility are accurately computed. A temperature and concentration-dependent power-law viscosity model in line with the experimental measurement of the fluid rheology is adopted. The model is first applied at isothermal condition to 2 benchmark samples, namely Fontainebleau sandstone and Grosmont carbonate, and is found to be in good agreement with the Lattice Boltzmann method (LBM). Finally, at non-isothermal conditions, an effective mobility is introduced that enables to perform a numerical sensitivity study to fluid rheology, heat transfer, and operating conditions. While the mobility seems to evolve linearly with polymer concentration in agreement with a derived theoretical model, the effect of the temperature seems negligible by comparison. However, a sharp contrast is found between carbonate and sandstone under the effect of a constant temperature gradient. Besides concerning the flow index and consistency factor, a master curve is derived when normalizing the mobility for both the carbonate and the sandstone.

## 1. Introduction

Understanding reservoir rock and fluid properties is essential for applications such as oil and gas industry, water management, hydrology, and geosciences. Oil exploration and production in conjunction with the prospective impact on the environment is highly related to the fluid flowing inside the highly complex geometry of the porous media [1,2]. In order to optimize reservoir management, the fluid flow processes in porous media should be investigated through a multiscale approach ranging from the field to the core level, down to the pore scale. One of the most important petro-physical properties for reservoir rock is the permeability, a measure of the flow capacity of the pore network. It is function of the complex microstructure of the rock, fluid properties (density, viscosity) and parameters (velocity). Since no simple universal correlation exists for the permeability, an accurate and efficient numerical tool to predict the permeability is highly desirable [2,3,4,5].

Using the 3D micro-CT images, numerical simulation has been applied to perform the pore-scale imaging modelling ranging from (i) the widely used lattice Boltzmann method [6,7]; (ii) the finite difference method [5]; (iii) finite element method [3]; and more recently (iv) finite volume method approach [4]. Most of the numerical simulation concerns with the modeling at pore-scale deals with Newtonian fluid or simplified pore network, which significantly alters the porous media geometry, assuming it consists of connected capillary tubes [8,9]. Despite a large body of work, modeling of non-Newtonian is still challenging and remains an active field of research [8,10,11,12,13]. Furthermore, the effect of the temperature-dependent viscosity is still to be addressed at the pore scale.

However, it is worth mentioned some interesting contributions of non-Newtonian fluids at Darcy scale both at isothermal and non-isothermal conditions. Using a rheological power-law fluid [14], a One-dimensional model of pressure front diffusion in a generalized geometry such as plane, radial, and spherical is derived. The pressure effect is investigated based on the derivation of a self-similar solution. The rate of advancement of the pressure front, as a function of the model parameters is also discussed in details. In [15] a validation of the theoretical prediction for the gravity currents of power-law fluids, intruding into a saturated porous medium, is provided. In addition, non-Newtonian power-law fluid has been adopted to numerically investigate the flow in rough-walled fractures [16,17], and an extension to a radial flow is proposed in [18]. The thermal effect at Darcy-scale has also been investigated where the consistency index of power-law fluid is considered to be temperature dependent [19]. In [10] thermal convective instability is carried out in 2D to identify the parameters that control the onset of thermally driven convection.

Predicting special core analysis (SCAL) data by means of digital rock physics (DRP) has gained attention recently. Most of the DRP simulations focus on Newtonian fluids and overlook the rheology of the fluid, which can be controlled by both the temperature and the polymer concentration. However, in petroleum engineering many fluids such as heavy oil and polymer solutions used for enhanced oil recovery (EOR) are non-Newtonian while the flow is non-isothermal at reservoir conditions. In order to use Digital Rock Physics (DRP) as a future tool to generating accurate, fast, and cost effective SCAL properties to support reservoir characterization and simulation; pore-scale modeling of non-Newtonian fluid at non-isothermal conditions is essential. This work proposes to shed a light on that aspect of the DRP.

In the present paper, we use a finite volume method (FVM) coupled with an adaptive mesh generation technique to perform the pore scale simulation from the micro-CT images of both a sandstone and a carbonate rocks from the literature. Besides, we performed simulations based on the LBM method for comparison and validation of the FVM on Newtonian fluid. Finally, we implement a power-law (or Ostwald deWaele) non-Newtonian fluid at non-isothermal conditions to test the pore-scale model sensitivity to both the rheological and operating parameters. This paper, focused on modeling at pore-scale, can be considered as complemental to the large body of works on simulating non-Newtonian fluids at Darcy-scale [14,18,20].

The paper is organized as follows: in Section 2, the governing equations are presented and in Section 3 the pore-scale modeling and validation are provided. Numerical results and sensitivity studies are performed in Section 4. Finally, conclusions are drawn in Section 5.

## 2. Pore-Scale Governing Equations

### 2.1. Mass and Momentum Conservations

The numerical simulation of a laminar flow inside a rock at pore-scale is considered. The fluid is assumed to be an incompressible non-Newtonian liquid. The continuity and momentum equations to be numerically solved in the finite volume method (FVM) formulation expresses as follows:

(1) ∇·V=0

(2) ρV∇V=−∇p+ρg+∇·τ

where V is the fluid velocity vector, and g denotes the gravity, while the fluid is assumed incompressible of density ρ. The stress tensor τ, assuming the viscosity depends on both the polymer concentration (*C*) and temperature (*T*), can be written as function of the shear rate ($\dot{\gamma }$) as follows:

(3) $$\tau =\mu \left(T,C,\dot{\gamma }\right)\left[\nabla V+\nabla V^{T}\right]$$

where μ is the viscosity which is assumed to be function of both concentration and temperature in addition to the dependency to the shear rate.

It is worth noting that the flow within the porous rocks is considered very slow and laminar, Reynolds number Re≪1, therefore the inertial forces can be neglected, and the momentum equations reduced to stokes Equation (2).

### 2.2. Energy Conservation

In addition to the mass and momentum conservation, we solve the energy conservation equation, given below:

(4) $$\rho C_{p}\nabla \;\cdot \;\left(VT\right)=\nabla \;\cdot \;\left(\lambda T\right)$$

where λ is the thermal conductivity and $C_{p}$ is the heat capacity. For simplicity we neglect the viscous dissipation since the Brinkman number, $Br=\mu U^{2}/\kappa \Delta T\ll 1$, of the laminar flow withing the porous rock is small.

### 2.3. Temperature-Concentration Power-Law Viscosity Model

The principle of polymer flooding is to increase water phase viscosity by adding polymer at a given temperature. For our modeling purposes, the resulting polymer solution is assumed homogeneous with a constant concentration which remains constant during the fluid flow through the porous media. In addition, polymer molecules’ size is assumed to be much smaller than the resolved pore-size (>2 μm), so that the flow can still be described under the continuum hypothesis (continuum flow region). The viscosity variation function of the polymer concentration (*C*) for EOR at different shear rate is evidenced in [21]. The fluid rheological behavior can be approximated over a limited range of shear rates by a simple non-Newtonian power-law fluid:

(5) $$\mu =\kappa \left(C,T\right){\dot{\gamma }}^{n-1},$$

where κ and *n* denote the consistency factor and the flow index, respectively. In addition, we assume that the consistency factor κ depends on both the temperature (*T*) and the polymer concentration (*C*). Besides $\kappa \left(T,C\right)\approx \mu_{T}\mu_{C}$ as the the concentration and the temperature (to a lesser extent for EOR polymers) are assumed to control independently the fluid viscosity.

At a fixed shear rate [21], the viscosity can be approximated by an exponential model as follows:

(6) $$\mu_{C}\propto \exp\left(\alpha C\right)$$

where α is a constant.

On the other hand, the effect of temperature on the viscosity for a liquid can be described by Guzman-Andrade equation derived by Eyring [22,23]:

(7) $$\mu_{T}\propto \exp\left(E/RT\right),$$

which has the same form as Arrhenius molecular kinetic equation. *E* is the apparent activation energy while RT is the thermal energy.

We can recast Equation (7) to introduce the ambient or reference temperature, $T_{\mathrm{ref}}$, so that the thermal effect cancels out when operating at ambient conditions in which most the experiments are performed:

(8) $$\mu_{T}\propto \exp\left[-\beta (T-T_{\mathrm{ref}})\right],$$

where β is an adjusting constant. Other temperature dependent viscosity models can be found in [10,19] in the context of simulation at Darcy-scale.

Finally, by adopting a two-parameters Ostwald-DeWaele non-Newtonian fluid and combining Equations (6) and (8) into Equation (5), we arrive at the following temperature and concentration dependent power-law model:

(9) $$\mu =\chi \exp\left[\alpha C-\beta (T-T_{\mathrm{ref}})\right]{\dot{\gamma }}^{n-1}$$

where *C* is the polymer concentration, $T_{\mathrm{ref}}$ the reference temperature, α is a constant, χ is the consistency factor and *n* is the flow behavior index.

**Figure 1:**
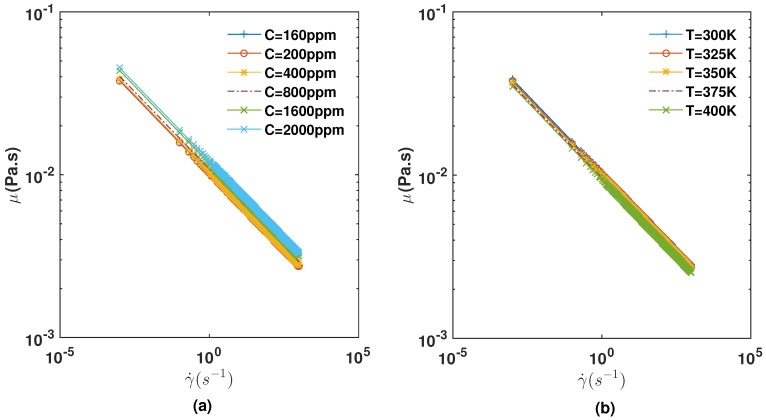
Temperature-Concentration Dependent Power-Law Viscosity Fluid Function of the Shear Rate at Different (**a**) Concentrations and (**b**) Temperature from Equation (9).

Equation (9) can be used to simulate many polymer solutions of interest to the EOR, and the constants can be found through the experimental measurements of the fluid properties. Furthermore, the effect of polymer concentration on the shear thinning behavior of bio-polymers seems to be well-captured by the proposed model equation given in Equation (9). This model (see Figure 1) is in line with experimental measurements of polymer solutions used for EOR [21,24,25]. Both the effect of temperature and concentration on the viscosity of polymer solutions are qualitatively well retrieved. The typical values used in Equation (9) are given in Table 1. It is worth noting that even though our model may approximate well some polymers rheology for EOR, generally polymer solutions exhibit a more complex constitutive relation such as in the case of viscoelasticity.

Due to the challenge to perform experiments at the scale of the micro-CT 3D images resolution and the characterization of fluid flow within the pore network, the present work will be based on numerical simulations to gain insight into the physics of modeling complex fluid through a rock.

## 3. Numerical Approach and Validation

**Figure 2:**
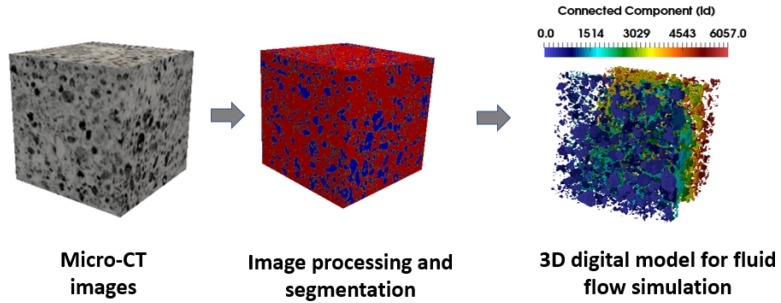
Workflow of the numerical simulation at pore-Scale of the micro-CT image of the rocks.

The workflow from the micro-CT image to the computation of petrophysical properties such as the permeability is summarized in Figure 2. The rock is first scanned at high resolution then segmented to discriminate between pore and solid phases; then the connectivity is determined in order to access the relevance of the numerical simulation. Finally the 3D digital rock model, the input of the numerical model, is generated.

One of the challenges in simulating flow in porous rocks is that experimentally, laboratory measurements cannot be performed at the scale in which pore phase is resolved. However, pore-scale modeling within the emerging digital rock physics (DRP) paradigm has gained attention recently going from understanding the physics within the rock to a predictive tools to support laboratory measurements [1,26]. The novelty of the present work is the development of a solver capable of computing at pore-scale non-Newtonian and non-isothermal fluid through porous rocks and gains insight into the flow behavior at scales unattainable experimentally. The pore-scale solver which can be used for different rock types and fluids can help in predicting flow behavior at reservoir conditions as well as to better understand polymer flooding.

**Figure 3:**
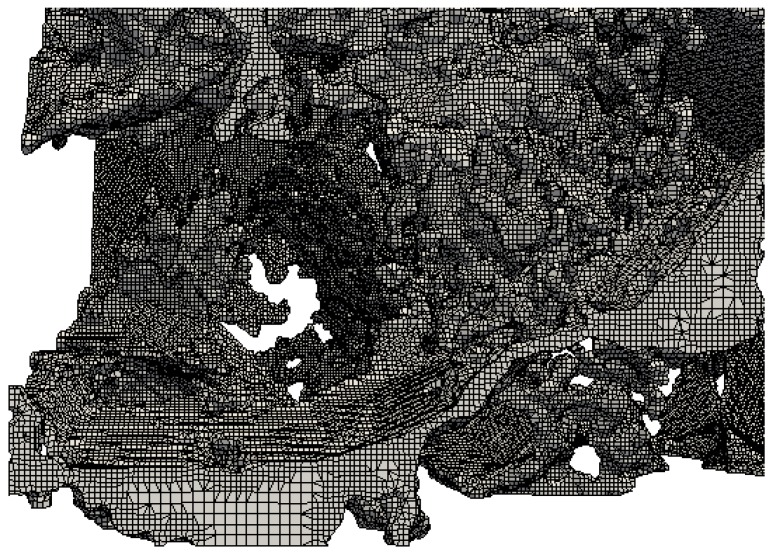
Pore-scale mesh generated from the digital image of the rock.

Before running the simulation, the segmented micro-CT image is meshed using SnappyHexMesh/ C++ code by employing an adaptive mesh generation technique, through refinement and adjustment to fit onto the provided geometries of the rock. Besides incorporation of boundary layers’ cells near the solid surface is also performed for better accuracy (Figure 3).

**Figure 4:**
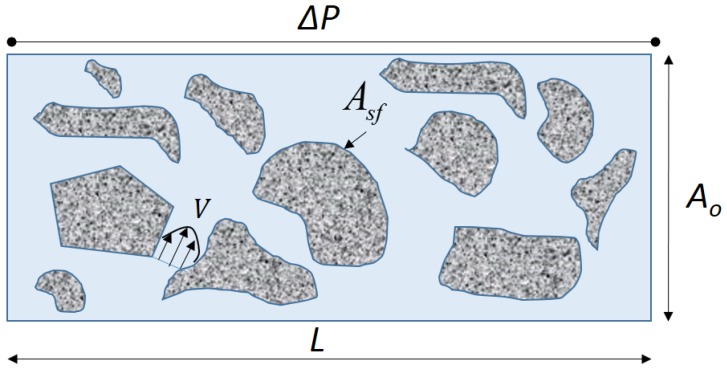
A schematic of the flow configuration at pore scale.

The pore-scale model is simulated by imposing a non-slip boundary conditions at the sides of the micro-plug sample and a pressure gradient between the inlet and outlet of the digital rock (Figure 4). The details on the boundary conditions used are provided in Table 2.

After solving the fluid equation, the permeability can be computed following the general Darcy’s law [27,28] as follows:

(10) $$\Delta P=-\frac{\mu (T)}{A_{o}^{n}K_{l}}Q^{n-1}LQ$$

where $A_{o}$ is the outlet surface area of the sample and $Q\equiv Q_{z}$ the *z*-component (horizontal direction) (see Figure 4) of the flow rate vector Q computed by integration from the outlet as

(11) $$Q=\int_{A_{o}}VdA.$$

Based on the homogenization theory in [29], Darcy equation for a non-Newtonian fluid such as the power-law fluid can be expressed as follows:

(12) $$\Delta P=-\frac{\mu_{\mathrm{eff}}LU}{K_{l}}=-\frac{\mu_{\mathrm{eff}}LQ}{K_{l}A}$$

where the effective viscosity, $\mu_{\mathrm{eff}}$, depends on the flow rate and porosity in addition to the fluid properties.

Due to the non-Newtonian nature of the fluid and the dependency of the viscosity to both temperature and concentration, and for convenience, we will adopt an effective fluid mobility ($M_{\mathrm{eff}}$), defined as the ratio between the effective viscosity and the permeability, to characterize the non-Newtonian fluid flowing inside the porous media defined as:

(13) $$M_{\mathrm{eff}}=\frac{K_{l}}{\mu_{\mathrm{eff}}}=-\frac{1}{\Delta P}\frac{LQ}{A}$$

where *Q* is the flow rate, ΔP the pressure gradient imposed on the sample, *L* and *A* are the sample length and surface area, respectively. It accounts for all the relevant parameters for the non-Newtonian flow in the porous media, namely the porosity, temperature, concentration, intrinsic permeability. It is worth noting that for a Newtonian fluid, n=1, the effective mobility reduce to $M_{\mathrm{eff}}=k/\mu$. For comparison with experimental data, we will use the mobility in dimensionless form by scaled it by the mobility of the Newtonian fluid, in the present case for water, $m_{\mathrm{eff}}=M_{\mathrm{eff}}/M_{\mathrm{effW}}$.

The governing equations Equations (1)–(9) are implemented in OpenFoam/C++ using second order linear upwind-biased schemes. To solve the discretized equations within the FVM framework, a Semi-Implicit Method for Pressure-Linked Equations (SIMPLE) algorithm is used to calculate the pressure and velocity fields using a Generalized Geometric-Algebraic Multi-Grid (GAMG) solver in conjunction with a Gauss Seidel smoother. The convergence criteria set for the pressure and velocity fields is of the order of 10${}^{-6}$. The simulation are performed on the two samples using the adaptive mesh technique consisting of 8,368,927 cells (8 millions) and 15,603,561 cells (15 milion) for the Fontainebleau sandstone and the Grosmont carbonate, respectively. The simulations are run in parallel using a domain decomposition method on 64 processors (4 nodes with 16 cores and 64 GB RAM each) with a typical cpu computation time of about 2 h.

### 3.1. Flow between Two Parallel Plates

We first validate the model for a Poiseuille-Hagen flow with both a Newtonian and non-Newtonian fluids flowing between two parallel plates. The non-Newtonian fluid viscosity is taken as a power-law and is given below:

(14) $$\mu \left(\dot{\gamma }\right)=\kappa {\dot{\gamma }}^{n-1},$$

for which an analytical solution can be written as:

(15) $$V_{x}\left(y\right)=\frac{n}{n+1}{\left(\frac{\Delta P}{\kappa L}\right)}^{1/n}\left[{\left(\frac{h}{2}\right)}^{1/n+1}-{\left|\frac{h}{2}-y\right|}^{1/n+1}\right]$$

or equivalently function of the flow rate *Q*,

(16) $$V_{x}\left(y\right)=\frac{Q}{B(n,h)}\left[{\left(\frac{h}{2}\right)}^{1/n+1}-{\left|\frac{h}{2}-y\right|}^{1/n+1}\right]$$

where $B\left(n,h\right)=2\left(n+1\right){\left(h/2\right)}^{2+1/n}/\left(2n+1\right)$. We performed the simulation at relatively low Reynolds number of, $Re=\rho V_{0}D_{0}/\mu =100$, to ensure a laminar flow.

**Figure 5:**
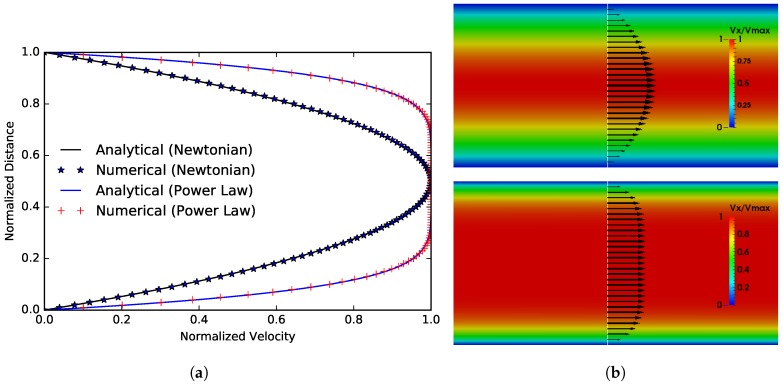
Comparison between numerical simulation and analytical solutions of the flow between two parallel plates, we set $\kappa =10^{-6}$ Pa.s${}^{n}$ and n=0.2 for the non-Newtonian fluid. (**a**) Axial velocity profiles; (**b**) Axial velocity vector and contour for the Newtonian (**top**) and Non-Newtonian (**bottom**) cases.

The comparison between analytical and numerical solutions is provided in Figure 5. A very good agreement of less than 0.5% the relative error is found.

### 3.2. Flow in Porous Media

**Figure 6:**
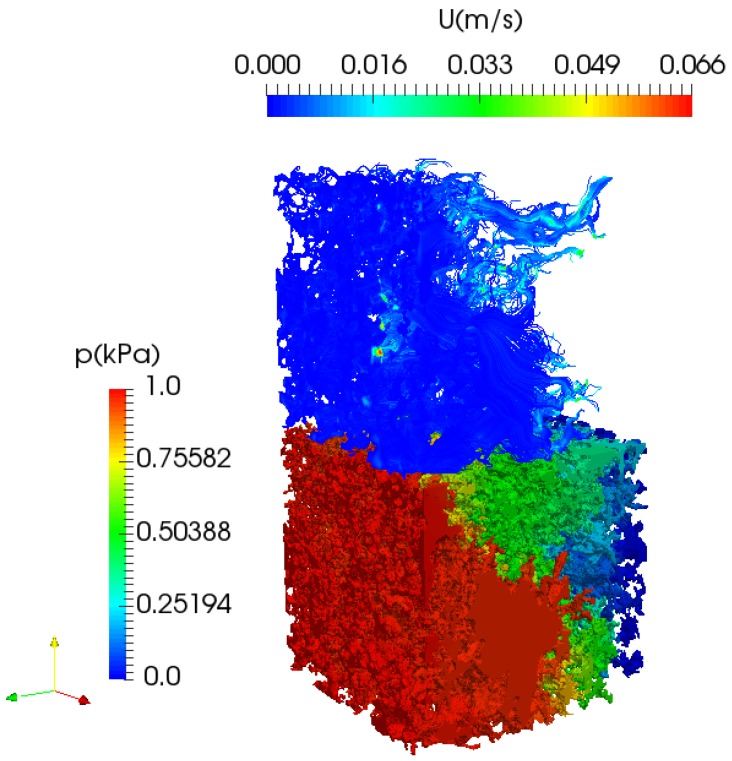
Simulated pressure field by FVM of the carbonate sample; streamlines, scaled by the velocity, are shown at the top of the pore structure for clarity.

For validation purpose of our model in porous media, we apply the FVM model to 2 rocks samples from the literature, the Fontainebleau sandstone and Grosmont carbonate [3]. We performed the simulations under the same conditions using the widely used LBM (Palabos library) from the literature. We provide in Table 3 the simulation results (Figure 6) of the absolute permeability along with the relative errors. For convenience the permeability is written in Darcy unit, 1 D = 9.869 233 $\times 10^{-13}m^{2}$ in line with the practice in the literature [4,5], the values in Table 3 are expressed in milliDarcy (mD), 1 mD = 10${}^{-3}$ D.

The difference is less than 2% suggesting that the implemented finite volume method (FVM) is capable to simulate accurately at the pore-scale. Unlike in the LBM, extension of our FVM model to non-Newtonian fluid can be handled without any numerical tuning parameters. Finally, although a good agreement is found with the implemented code, a full validation would required to perform pore-scale experiment within the same scanned rock, which experimentally is still to be addressed.

## 4. Pore-Scale Non-Newtonian and Non-Isothermal Fluid Flow Simulation

### 4.1. Effect of the Polymer Concentration on the Mobility

**Figure 7:**
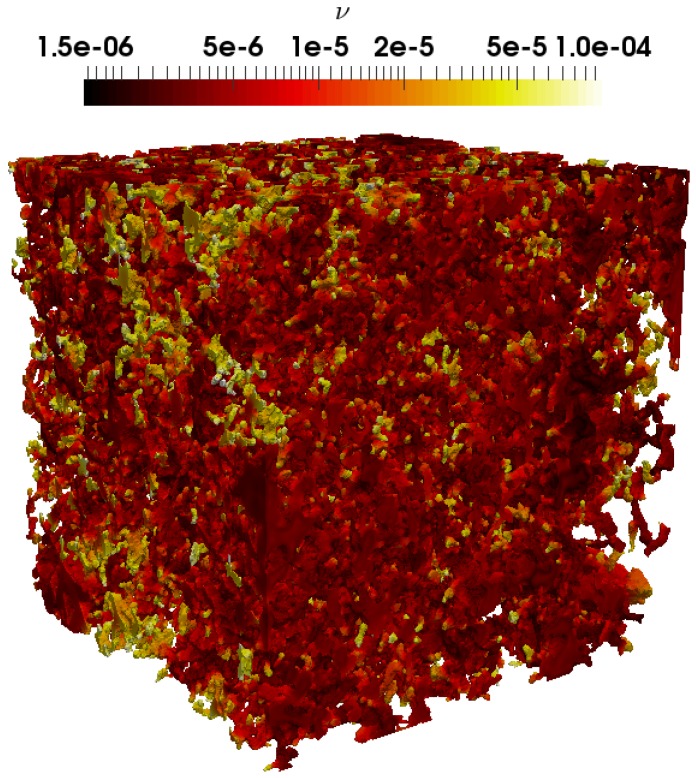
Simulated kinematic viscosity field ν (m${}^{2}$ s${}^{-1}$) for the carbonate, displaying in logarithmic field for clarity.

**Figure 8:**
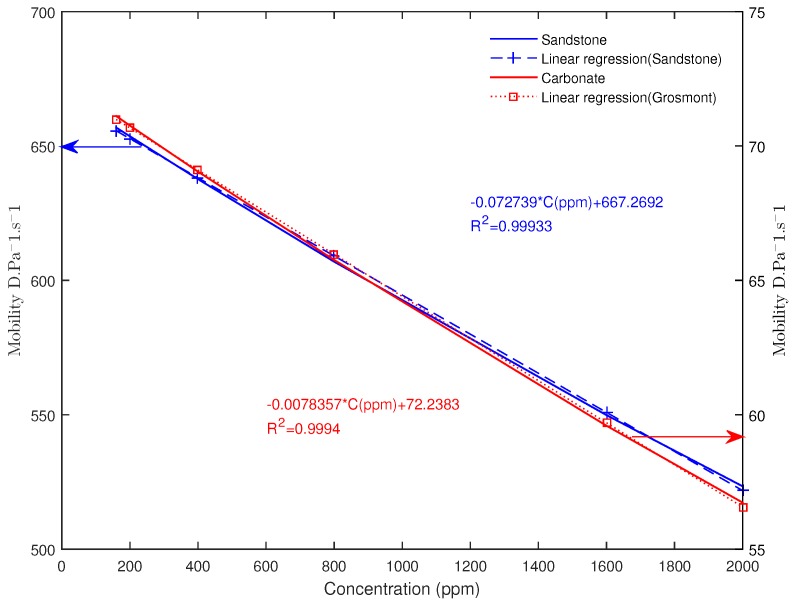
Simulated results of the variation of the effective mobility Equation (13) function of the polymer Concentration.

The mobility describes the ability of the porous rocks to transmit the fluid, in the present case a power-law non-Newtonian fluid. The high mobility would indicate a better transmissibility of the fluid as opposed to the low mobility. In order to investigate the concentration effect on the mobility, we simulated the fluid flow at pore-scale by varying the concentration at a fixed temperature using the proposed fluid rheology model given in Equation (9). The kinematic viscosity field resulting from the simulation, which spans two orders of magnitude, is shown in Figure 7, highlighting the complexity nature of the concentration-dependent shear-thinning fluid flow within the rock. The effect of the concentration on the mobility for both the carbonate and sandstone are given in Figure 8. As expected, the mobility seems to evolve inversely proportional to the concentration. Interestingly, while the viscosity exponentially depends on the concentration, the mobility seems to evolve linearly with it for both samples. In order to interpret the linear evolution of the mobility with the concentration, let’s assume that the fluid flow within the porous rocks follow a Poiseuille type flow. In this case, the flow rate can be related to the pressure gradient from Equation (15) to Equation (16) by

(17) $$Q\propto {\left(\frac{\Delta P}{\kappa }\right)}^{1/n},$$

where the flow consistency factor $\kappa =\mu_{C}\propto \exp\left(\alpha C\right)$, by neglecting the thermal contribution as $T=T_{\mathrm{ref}}$. Using the definition of the effective mobility in Equation (13) we have:

(18) $$M_{\mathrm{eff}}\propto \frac{LQ}{A\Delta P}\propto \frac{L}{A\Delta P}{\left(\frac{\Delta P}{\kappa }\right)}^{1/n},$$

as ΔP, *A* and *L* are fixed, we can simplify

(19) $$M_{\mathrm{eff}}\propto {\left(\frac{1}{\mu_{C}}\right)}^{1/n}\propto \exp\left(-\alpha C/n\right).$$

On the other hand since ϵ=αC/n≈0.2≪1 even by taking the maximum concentration *C* = 2000×10${}^{-6}$, α = 100, *n* = 0.81. Therefore, by expanding Equation (19) at the 1st order, we can deduce the following equation:

(20) $$M_{\mathrm{eff}}\propto 1-\frac{\alpha C}{n}$$

which predicts a linear variation of the mobility with respect to the concentration, in agreement with the 3D simulation of the 2 rocks samples. However the simplified model does not account for the pore structure.

### 4.2. Effect of Temperature Gradient on the Mobility

**Figure 9:**
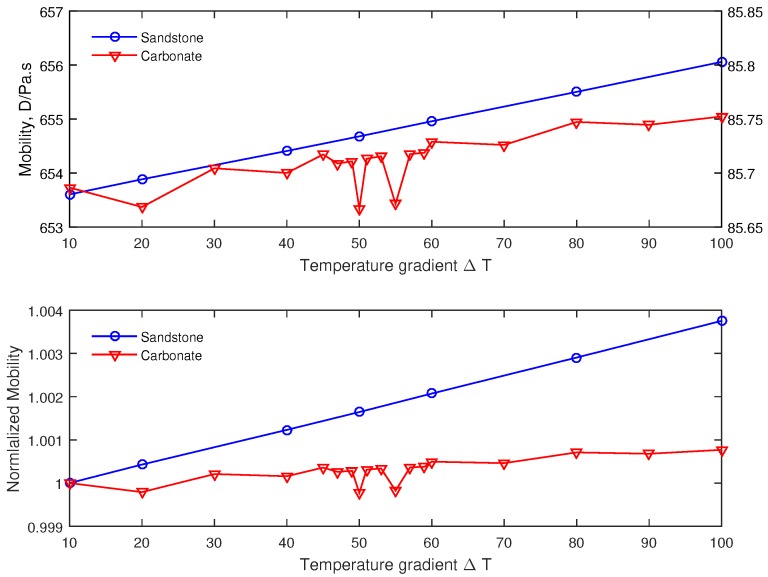
Variation of the effective mobility based on the temperature gradient imposed on the pore-scale model for the Fontainebleau sandstone and Grosmont carbonate.

We numerically impose different temperature gradients on the pore-scale model. The effect of temperature gradient seems to be very limited in controlling the mobility. However, an interesting evolution can be observed in Figure 9 highlighting the contrast between the two samples. Sandstone rock seems to follow a linear trend while the variation for the carbonate is non-monotonic. In fact, we can observe that the mobility dependency to the temperature gradient is complex, with trends difficult to predict. From the ambient condition an increasing of the temperature gradient leads to an irregular behavior of the mobility.Although the temperature effect is overall negligible on the mobility, there is a slight change in the flow pattern within the porous media. The permeability or mobility tensor is a global quantity, which is derived from the flow rate Q ($Q_{x}$, $Q_{y}$, $Q_{z}$) by integrating the velocity vector over the outlet Equation (11). In deriving the *z*-component, there are other components of the velocity vectors on the remaining directions (*x*,*y*), which are not account for when the mobility or permeability is computed over the *z*-axis. The difference in the mobility over the temperature gradient range is less than 0.1% (Figure 9). This is in the same range as the variation of the transversal components of Q about 0.08% of $Q_{z}$. Ultimately, the slight difference in the outlet velocity vectors orientation could explain the irregular variation (within 0.1%) of the mobility observed in Figure 9. However, this behavior seems to suggest that when dealing with non-Newtonian fluid in complex pore structured rock, such as carbonate, there may be an optimal temperature gradient to obtain higher mobility. A consequence of this finding may help in determining adequate operating conditions during oil production from wellbore where temperature gradient comes into play.

### 4.3. Effect of the Fluid Rheological Parameters on the Mobility

**Figure 10:**
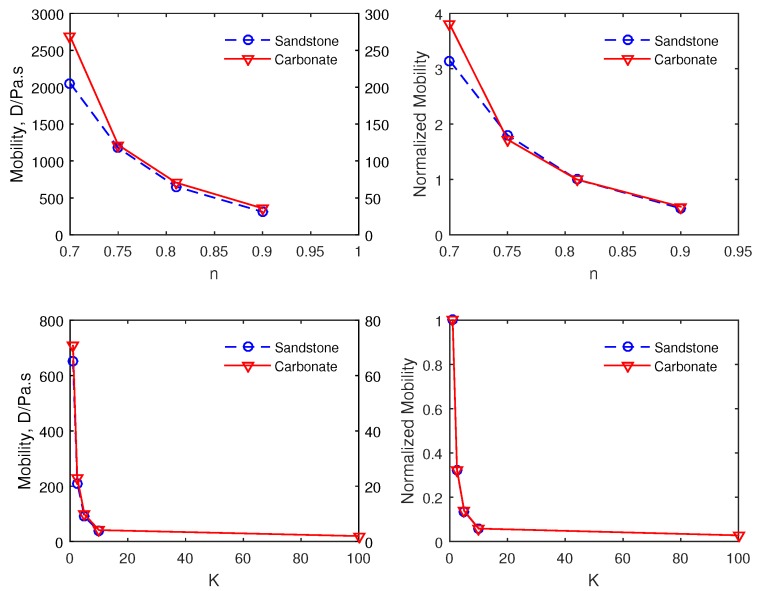
The sensitivity of the effective mobility to (**top**) the flow behavior index n and (**bottom**) the consistency factor (K) at a temperature of 300 K and 200 ppm of the polymer concentration. The effective mobility is being normalized with that of water as detailed in Section 2.

We numerically investigate the sensitivity of the model to the rheological properties of the fluid, namely the flow behavior index *n* and the consistency factor *K*. We depict in Figure 10 the impact of both *n* and *K* on the mobility.

We observe that the mobility is reduced by an increase of both *n* and *K*. Even though, we have similar trends for both carbonate and sandstone on the fluid consistency factor (*K*), there is however a slight difference on the behavior index (*n*). In addition, the effect of *K* on the mobility seems to be more pronounced than that of *n*. It is worth noting the challenge posed experimentally to discriminate the effect of these two parameters contribution on the mobility. Hence, the possibility to perform such a sensitivity study numerically can help in the formulation of polymer solutions for EOR.

### 4.4. Effect of the Pressure Gradient

**Figure 11:**
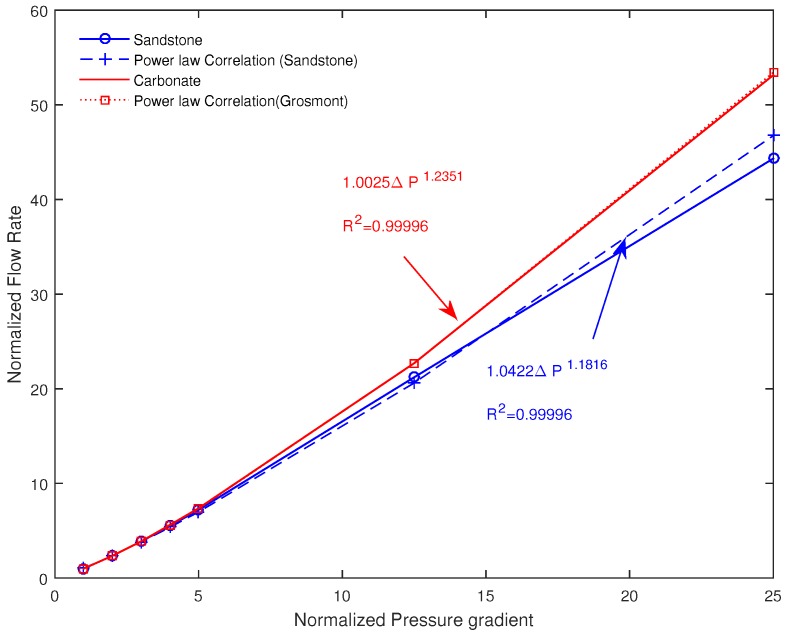
Simulated normalized flow rate and pressure gradient variation from pore-scale non-Newtonian model.

Here the model sensitivity under the effect of pressure gradient is investigated from the two samples, namely the Fontainebleau sandstone and the Grosmont carbonate. In Figure 11, we provide the simulation results where the evolution of the normalized flow rate and pressure gradient. We found that the results can be correlated to the following power-law, $Q/Q_{\mathrm{Newtonian}}\approx \Delta P^{1/\xi }$. Interestingly, the parameter ξ evolves close to *n*, the flow behavior index. The non-linearity nature of the fluid flow within the porous media seems to be more dominant for the carbonate, of complex structure, than for the sandstone sample. This effect is more pronounced at higher pressure gradient and ξ seems be a good parameter to reveal pore structure complexity. Remarkably, the dependency of *Q* on the power of pressure gradient is in agreement with the recent founding experimentally at Darcy scale in [30] for yield stress fluid and in [20] for Herschel-Bulkley fluid in the context of gravity current.

## 5. Conclusions

A comprehensive numerical model based on the Finite Volume Method (FVM) and an adaptive meshing technique to describe the flow properties at pore-scale of a non-Newtonian fluid is presented. The fluid rheology is modeled by incorporating its dependency to the concentration and temperature into a generalized power-law viscosity fluid model. Based on Newtonian fluid through rock samples from the literature, the FVM algorithm is validated against a Lattice Boltzmann Method (LBM). After implementing the non-Newtonian and non-isothermal fluids, the model is validated by simulating the fluid flow between two parallel plates for which the analytic solution is known. Subsequently, the model sensitivity to heat transfer and fluid rheology is tested by evaluating the effect of polymer concentration on the mobility as well as the relationship between flow rate and pressure gradient. The effective mobility dependency on the polymer concentration follows a linear trend, in line with a derived theoretical model, while the flow rate displays a disparity between the carbonate and sandstone. Besides the fluid rheological properties such as the flow behavior index and consistency factor lead to a master curve when normalizing the mobility for both the carbonate and the sandstone. Overall, the present work serves to implement a new simulator capable of computing the permeability and mobility of non-Newtonian fluid based on its thermal and rheological properties. The solver which can be used for different rock types at pore-scale can provide valuable insight into predicting flow behavior at reservoir conditions as well to better understanding polymer flooding for enhanced oil recovery (EOR).

## Figures and Tables

**Table 1:** Typical values of the viscosity model parameters.

Parameters	χ (Pa s${}^{n}$)	α (-)	β (K${}^{{}^{-1}}$)	Tref (K)	*n* (-)
Values (SI)	10${}^{-2}$	100	10${}^{-3}$	293	0.81

**Table 2:** Boundary conditions.

Boundaries	Pressures	Velocity	Temperature
Inlet	fixed value, P = 1000 Pa	normal gradient, $\frac{\partial V}{\partial n}$ = 0	fixed value, T
Outlet	fixes value, P = 0 Pa	normal gradient,$\frac{\partial V}{\partial n}$ = 0	fixes value, T = 300 K
Sides	normal gradient, $\frac{\partial p}{\partial n}$ = 0	fixed valued, **V** = 0	normal gradient, $\frac{\partial T}{\partial n}$ = 0
Pore/rock interface	normal gradient, $\frac{\partial p}{\partial n}$ = 0	No-slip, **V** = 0	normal gradient, $\frac{\partial T}{\partial n}$ = 0

**Table 3:** Numerical Simulations Results of the Absolute Permeability in *z*-axis.

Sample	Image Size	Voxel Size (μm)	Porosity (%)	FVM (mD)	LBM (mD)	Relative Errors (%)
Fontainebleau Sandstone	288×288× 300	7.5	14.5	1614	1610	0.2
Grosmont Carbonate	400×400× 400	2.02	24.7	217	214	1.4
